# Violence at Work and Mental Distress among Firefighters in Guatemala

**DOI:** 10.29024/aogh.2306

**Published:** 2018-10-10

**Authors:** Claudia Meneses Pinto, Katja Radon, Frank van Dijk

**Affiliations:** 1 Center for International Health @ Institute for Occupational, Social and Environmental Medicine, University Hospital Munich (LMU), DE; 2 LDOH — Learning and Developing of Occupational Health, Loosdrechtse Bos 17, 1213 RH Hilversum, NL

## Abstract

**Background:**

Firefighting is a highly physically and mentally demanding occupation. In many countries, firefighters are frequently exposed to critical events, violent threats and assault in their job, however, knowledge about its prevalence is limited. In addition, little is known about the impact of workplace violence against firefighters in the development of mental distress.

**Objective:**

We aimed to determine the prevalence of mental distress in firefighters exposed to workplace violence.

**Methods:**

In this cross-sectional study, 141 male full-time firefighters of Guatemala City and Metropolitan Area (response 80.4%) were invited to answer an interview-based questionnaire including items on sociodemographics, working conditions and mental health (general health questionnaire GHQ-12). Mental distress was defined as a GHQ-12 score above 4. The item ‘12-months prevalence of violence on the job’ included physical violence or sexual abuse from colleagues or the public. Statistical analysis with Epiinfo 7 included descriptive, bivariate and logistic regression analyses.

**Findings:**

Exposure to violence at the workplace was common (37%). Prevalence of mental distress was higher in violence-exposed firefighters (54%) than among unexposed firemen (39%; p = 0.08). After adjustment, the odds for distress was not statistically significantly increased for those exposed to violence at the workplace in comparison to the unexposed group (1.87; 0.90–3.87). Especially affected by distress were middle-aged firefighters (40–49 years; adjusted Odds Ratio 2.90; 95% Confidence interval 1.20–7.05) compared to younger firemen (<40 years).

**Conclusions:**

Exposure to violence at the workplace is common in Guatemalan firefighters. Although limited by small numbers, the association between violence and poor mental health is plausible. Therefore, training programs strengthening resilience among firefighters in areas with high crime prevalence are warranted.

## Introduction

Firefighting is a highly dangerous and stressful occupation, risking the firefighter’s own life [1] while sometimes being exposed to traumatic events and critical incidents [2]. Besides being exposed to such hazards inherent to the occupation, firefighters might also be exposed to workplace violence, such as assault, and verbal and physical threats from the public, including bodily harm from alcoholics and drug addicts [2]. In general, internal workplace violence or harassment, lacking a standard definition, covers all kinds of abuse inside the job, while the related concept of ‘external workplace violence’ refers to threats, physical or psychological violence carried out by third parties, such as patients or public [3].

Studies demonstrate several consequences of the combination of stressful events and emotional demands among firefighters, such as Post Traumatic Stress Disorder (PTSD) [1,2,4,5,6], mental health complaints [4,7,8,9,10,11], substance abuse (alcohol, nicotine, caffeine) [12,13], work related musculoskeletal disorders [14], as well as injuries and accidents [11].

Among these consequences, probably the most studied one in firefighters is PTSD, an anxiety disorder with high prevalence among firefighters and other emergency service workers [2,6]. Well known in this context are the studies carried out after the World Trade Center tragedy in the USA, where the prevalence of PTSD was estimated to be between 4% and 20% among involved rescue workers [5]. Outside the World Trade Centert tragedy, the prevalence among firefighters was 15–20% in Denmark [4] and 21% in Iran [6]. Other studies indicated that between 49% and 70% of firefighters suffer from sleeping problems [8,9,13]. Sleeping problems might result in a cycle of insomnia, tension, fatigue, depression, lower job performance and even higher levels of distress [7,8]. Alcohol dependence and other substance abuse might be associated with sleeping disorders, high job demands and low job control, and depression in firefighters [10,12,13,15]. At the same time, an unusual resilience was described in this group. High social support and low levels of self-blame seem to contribute to resilience among firefighters [5].

To our knowledge, few studies focus on mental health problems other than PTSD in firefighters [7,8]. The mental health of firefighters might especially be affected in high crime areas were exposure to violence at work is frequent. Guatemala is one of the most violent countries in the world, with an annual rate of 47 homicides per 100,000 habitants in 2006, decreasing to 7 homicides per 100,000 habitants in 2016 [16,17]. The constant exposure to violence generates distress with potential effects on the population’s mental and physical health [16]. The Guatemalan “*Benemérito Cuerpo Voluntario de Bomberos de Guatemala*” (Volunteer Firefighter Department of Guatemala) is an autonomous public service entity, with approximately 5,000 members, both volunteers and full-time-employees, providing services such as attending to medical emergencies and rescues, as well as common fires, and industrial and forest fire control. Little is recorded about the mental health of firefighters in Guatemala and no information is available about the mental health effects of their exposure to violence at work. Given this situation, the objective of the study was to determine the prevalence of psychological distress in full-time Guatemalan firefighters and its association with the exposure to violence at work. The results of this study may be used to develop and implement firefighters’ mental health prevention and surveillance programs.

## Methods

### Study population

This cross-sectional study was conducted in December 2013 and January 2014 on firefighters of the “Benemérito Cuerpo Voluntario de Bomberos de Guatemala”, which is composed both of the “permanent guard” (full-time paid employees) and volunteer members (part-time, not paid). The requirements to become a member are being between 18 and 38 years of age, to be approved for a 10-month training course, and to be at optimum physical and mental health conditions. However, there are no formal pre-employment or periodical health surveillance programs offered to would-be membres. Due to legal issues, their employment conditions are not clearly defined. The age of retirement, although established at 62 years or more than 20 years of service, is not fully implemented. Based on data from 2013, currently not even one firefighter has received the benefits of retirement; that is the reason why they continue to work beyond the age of retirement.

Of the 5000 members, 544 were members of the permanent guard. Of those, the 259 firefighters working in Guatemala City and nearby departments formed our sampling frame. Of the 200 randomly selected firefighters, 152 participated in the study (response 80.4%). They were working in one of the 24 firefighter stations, of which 15 are located in Guatemala City and 9 outside the metropolitan area. Reasons for non-participation were presumably lack of internal communication about the study in some stations and lack of interest in the study.

### Study Instruments

The Working and Health Conditions in Latin America Survey was used for this study, which was developed by an international expert team of the Center for International Health at the Ludwig-Maximilians-University, Munich, Germany. It consists of items taken from different studies on working conditions and health in their Spanish version [18,19,20,21,22].

Ethical approval was obtained from the Ethical Committee of the University Hospital of Munich, Germany as well as from Zugueme, a recognized private local committee in Guatemala. The field work coordinator invited the firefighters, via their Human Resources Manager, to participate. The participation was voluntary and anonymous, after obtaining informed consent. Two trained interviewers conducted personal interviews.

### Data entry and definition of variables

We completed a double data entry method using EpiInfo Version 7 with error correction. Hard copies of questionnaires were filed for quality control.

Exposure to violence at work during the 12 months prior to the survey was considered present if the firefighter answered affirmative to any of three questions on internal physical violence, external physical violence (e.g. by patients, relatives) or sexual harassment. The outcome was defined using the 0011 scaling method of the General Health Questionnaire. Firefighters with a total score of 5 or above were considered to suffer from mental distress [23].

With respect to potential confounders, age was combined into three categories: <40 years, 40–49 years, and ≥50 years. Educational levels were divided into basic or elementary education, middle or high school degree, and university level. Years of service were dichotomized to <five years and ≥five years. Based on the location of the company, we distinguished between those who were located inside or outside the Department of Guatemala City. Firefighters were divided into those only working as firefighters and those having more than this job. Weekly working hours were dichotomized into <40 vs. ≥40 hours.

### Statistical Analyses

The statistical analysis was done with Epiinfo™ Version 7. Due to small sample size (N = 11) women were excluded, leaving 141 male firefighters for analyses. Descriptive analyses were conducted comparing the relative frequencies of potential confounders of firefighters who did and those who did not suffer from violence at the workplace during the 12 months prior to the survey. The prevalence of mental distress was stratified for potential confounders. Statistical independence was tested by Chi-square test with two-tailed *p*-values. Those variables with *p < 0.10* were included in the multivariate logistic regression model analyzing the association between the exposure to violence at work and psychological distress.

## Results

The study population of 141 male firefighters was young, with almost half of the participants (48%) younger than 40 years and 26% 50 years or older. They mainly possessed a middle or high school degree. With respect to employment conditions, the majority reported an employment duration of more than 5 years (84%), working more than 40 hours per week (80%) and working only as a firefighter (82%).

The 12-month prevalence of any violence at work was 37%. Most common was external workplace violence (31%), followed by internal workplace violence (12%) and sexual harassment (4%). Sociodemographic factors and employment conditions did not differ between those reporting exposure to any type of violence and those who did not (Table 1).

**Table 1:** Sociodemographic data and employment conditions stratified for 12-months prevalence of physical violence at work among 141 firefighters working in Guatemala.

Variable	Categories	N_missing_	Exposure to physical violence at work		pChi^2^

			Yes^1^ N = 52	No^1^ N = 89	

			%	%	

**Age**	<40 ys	0	44.2	50.6	0.93
	40–49 ys		25.0	25.8	
	50+ ys		30.8	23.6	

**Educational Level**	Basic or Elementary School	0	21.2	29.2	0.43
	Middle or High School		61.5	50.6	
	University		17.3	20.2	

**Hours worked per week**	<40	5	16.0	19.8	0.58
	≥40		84.2	80.2	

**Duration of employment**	<5 years	6	13.7	17.9	0.53
	≥5 years		86.3	82.1	

**Location of fire station in Guatemala**	Outside Guatemala Department	1	28.9	27.3	0.84
	Within Guatemala Department		71.2	72.7	

**Number of jobs**	One	0	82.7	80.9	0.79
	More than 1		17.3	19.1	

^1^ Exposure to violence defined as 12-month prevalence of exposure to physical violence at work, either by persons belonging or not to the workplace (e.g. patients), and/or sexual harassment.

The overall prevalence of mental distress was 44%. Those reporting exposure to violence at the workplace had a non-statistically significantly higher prevalence of mental distress (54%) than those without (39%; p = 0.08). The prevalence of mental distress was highest among firemen in the age group 40 to 49 years (66%) as compared to younger (37%) and older (38%) firefighters (p = 0.01). None of the other sociodemographic variables or employment conditions was associated with mental distress (Table 2).

**Table 2:** Prevalence of mental distress by sociodemographic factors and employment conditions among 141 firefighters working in Guatemala.

Variable	Categories	Mental distress (GHQ-12 Score ≥5)				

		N_missing_	%	pChi^2^	Crude Odds Ratio (95% Confidence Interval)	Adjusted Odds Ratio (95% Confidence Interval)

**Violence at the workplace**	No	1	38.6	0.09	1	1
	Yes		53.9		1.88 (0.89–3.96)	1.91 (0.87–4.18)

**Age**	<40 ys	1	36.8	0.01	1	1
	40–49 ys		65.7		3.30 (1.40–7.75)	2.90 (1.20–7.05)
	50+ ys		37.8		1.05 (0.46–2.39)	0.78 (0.32–1.89)

**Educational level**	Basic or elementary school	1	51.4	0.51	–	–
	Middle or High school		43.4		–	–
	University		37.0		–	–

**Hours worked per week**	<40	6	44.0	0.97	–	–
	≥40		43.6		–	–

**Duration of employment**	<5 ys	1	31.8	0.16	–	–
	≥5 ys		48.2		–	–

**Location of fire station**	Outside Guatemala Department	0	47.4	0.69	–	–
	Within Guatemala Department		43.6		–	–

**Number of jobs**	One	0	44.7	0.82	–	–
	More than 1		42.3		–	–

– Variables not considered for the logistic regression models because pChi^2^ was above 0.10 in the bivariate analyses.

The logistic regression analyses confirmed these findings. While violence was not statistically significantly associated with increased odds of mental distress (Odds Ratio 1.87; 0.90–3.87 compared to unexposed firemen), the mutually adjusted odds for firemen in the age range 40 to 49 years was statistically significantly increased compared to younger participants (2.90; 1.20–7.05; Table 2).

## Discussion

This cross-sectional study of Guatemalan firefighters indicates that exposure to violence is common. At the same time, prevalence of mental distress was shown to be high. Results suggest an association between exposure and outcome, albeit statistical analyses did not reach the level of statistical significance.

The prevalence of distress found in our study was higher than the one in a meta-analyses of mental distress among UK service personnel deployed to war areas – even though they defined distress as a GHQ-12 score of 4 and higher [24] while our cut-off was 5 or higher. Comparing our results to other professions, the prevalence of mental distress among men was 14% in male Peruvian offshore workers, 20% in Chilean informal miners, 29% in Peruvian formal miners and 82% in Bolivian cooperative miners using the same cut-off point as in this study [25]. No study using the same instrument to assess mental distress and the same scoring method could be found for firefighters nor the Guatemalan general population. No national cut-off for the GHQ-12 based on national validation studies for Guatemala is available. Given large cultural differences in GHQ-12 results, data from other countries are difficult to compare to the Guatemalan society [26]. This is especially important as our study was carried out in one of the most violent countries in the world, recognizing that collective violence increases the risk of poor mental health in the general population [27].

Likewise, no other study among Latin American firefighters assessing exposure to violence on the job could be found. The 12-month prevalence of physical violence at the workplace in our study was 37%. In the US, a national survey including firefighters found a similar 12-month prevalence of external physical violence. Of these firefighters, 43.4% reported physical violence from patients while 5.8% reported physical violence from a patients’ family member [28]. In contrast, a study among male Dutch firefighters reported a 12-month prevalence of external physical violence of only 13.8% [3]. Comparing the prevalence of internal physical violence (12%) and sexual harassment at the workplace (4%) among our study population to the general male Guatemalan working population (internal physical violence 4%; sexual harrassment 1%), the exposure frequency was much higher in our study population [29]. Unfortunately, no data on the prevalence of external physical violence in the general Guatemalan working population exists. However, our data indicate that this is the most frequent source of exposure in the firefighters.

We identified an age between 40 and 49 years as a risk factor for mental distress. Reason for this might be a healthy worker survivor effect [30,31]: while young firefighters are still able to cope with the situation, older firefighters are more affected by the psychosocial working conditions and develop mental health complaints. With increasing age, those affected leave the job, seeking other opportunities. This reasoning is supported by the fact that about half of our study population was younger than 40 years of age. However, health-based selection in firefighters most likely also includes physical health conditions.

Overall, our results confirm an association between exposure to physical violence and mental distress found in other studies [32,33]. The fact that the association did not reach the level of statistical significance is most likely due to a lack of statistical power resulting from small sample size combined with a high prevalence of violence and mental distress. Another limitation of our study is that we only considered physical violence so we cannot assess the prevalence of verbal violence or discrimination. In addition, we did not assess the type of violence suffered so we cannot distinguish between different kinds of violence. Exposure and outcome were self-reported, potentially leading to common method bias and thus spurious associations. The questionnaires were answered in interviews by well-trained interviewers; nevertheless, social desirability bias might led to an underestimation of exposure and outcome.

Among the strengths of the study are the high response (80%) which most likely reduced selection bias. However, it could be that some of the non-responders were affected by mental health problems. Furthermore, we used validated questionnaire instruments including potential confounders. Only full-time-paid firefighters were included in order to minimize the potential bias of adverse health effects from exposures at another or even the main job. Nevertheless, one-fifth of the firefighters held an additional job. However, holding a second job was neither associated with the exposure nor with the outcome of the study.

In conclusion, this study contributes to the evidence that firefighters in Guatemala are exposed to high levels of workplace violence, especially external violence, besides being exposed to critical incidents inherent to the occupation. As exposure to physical violence is associated with mental distress, intervention programs helping to increase the resilience of the firefighters are warranted.
